# Arrhythmogenic Substrates for Atrial Fibrillation in Obesity

**DOI:** 10.3389/fphys.2018.01482

**Published:** 2018-10-22

**Authors:** Ellen R. Lubbers, Morgan V. Price, Peter J. Mohler

**Affiliations:** ^1^The Dorothy M. Davis Heart & Lung Research Institute, The Ohio State University Wexner Medical Center, Columbus, OH, United States; ^2^Medical Scientist Training Program, The Ohio State University Wexner Medical Center, Columbus, OH, United States; ^3^Department of Physiology & Cell Biology, The Ohio State University Wexner Medical Center, Columbus, OH, United States; ^4^Department of Internal Medicine, The Ohio State University Wexner Medical Center, Columbus, OH, United States

**Keywords:** obesity, arrhythmia, atrial fibrillation, inflammation, remodeling, oxidative stress

## Abstract

Global obesity rates have nearly tripled since 1975. This obesity rate increase is mirrored by increases in atrial fibrillation (AF) that now impacts nearly 10% of Americans over the age of 65. Numerous epidemiologic studies have linked incidence of AF and obesity and other obesity-related diseases, including hypertension and diabetes. Due to the wealth of epidemiologic data linking AF with obesity-related disease, mechanisms of AF pathogenesis in the context of obesity are an area of ongoing investigation. However, progress has been somewhat slowed by the complex phenotype of obesity; separating the effects of obesity from those of related sequelae is problematic. While the initiation of pathogenic pathways leading to AF varies with disease (including increased glycosylation in diabetes, increased renin angiotensin aldosterone system activation in hypertension, atrial ischemia in coronary artery disease, and sleep apnea) the pathogenesis of AF is united by shared mediators of altered conduction in the atria. We suggest focusing on these downstream mediators of AF in obesity is likely to yield more broadly applicable data. In the context of obesity, AF is driven by the interrelated processes of inflammation, atrial remodeling, and oxidative stress. Obesity is characterized by a constant low-grade inflammation that leads to increased expression of pro-inflammatory cytokines. These cytokines contribute to changes in cardiomyocyte excitability. Atrial structural remodeling, including fibrosis, enlargement, and fatty infiltration is a prominent feature of AF and contributes to the altered conduction. Finally, obesity impacts oxidative stress. Within the cardiomyocyte, oxidative stress is increased through both increased production of reactive oxygen species and by downregulation of scavenging enzymes. This increased oxidative stress modulates of cardiomyocyte excitability, increasing susceptibility to AF. Although the initiating insults vary, inflammation, atrial remodeling, and oxidative stress are conserved mechanisms in the pathophysiology of AF in the obese patients. In this review, we highlight mechanisms that have been shown to be relevant in the pathogenesis of AF across obesity-related disease.

## Introduction

Obesity rates are increasing worldwide, nearly tripling in the last 40 years (Ncd Risk Factor Collaboration, 2016). Now, more than of a third of the population of the world is obese or overweight. In the United States, ∼38% of adults are considered obese, with a body mass index (BMI) over 30, and an additional 33% are considered overweight, with a BMI between 25 and 30 (Hales et al., 2017). Reflecting the broad impact of the obesity epidemic, rates of many obesity-related diseases have also increased (Hruby and Hu, 2015).

Many population-based studies have determined that obesity is a significant risk factor for arrhythmia, including atrial fibrillation (AF) (Wang et al., 2004; Wanahita et al., 2008; Goudis et al., 2015). Obesity has been identified as an independent risk factor for AF, with each 5-unit increase in BMI contributing an additional 20% risk of AF (Grundvold et al., 2015; Karam et al., 2017; Lavie et al., 2017). AF is the most common sustained arrhythmia in adults and with ∼6.1 million people affected in the United States (January et al., 2014). Briefly, AF is a reentrant arrhythmia characterized by the disorganized depolarization and contraction of the atria. In order to sustain a reentrant arrhythmia, three conditions are generally present: (1) the atria must be sufficiently electrically or structurally remodeled such that the tissue ahead of the wavefront is excitable. This may occur through reduced conduction velocity increased total path length, and/or decreased effective refractory period. (2) There must be unidirectional conduction block, preventing self-elimination of the wave when fronts meet each other. (3) There must be a physical or functional obstacle around which the reentrant wave rotates (Antzelevitch and Burashnikov, 2011; Tse et al., 2016). Thus, mechanisms contributing to the pathophysiology of AF in obesity do so by contributing to one or more of these three criteria.

However, the mechanistic links between obesity and AF are confounded by the complex pathophysiology of obesity. Obesity is characterized by increased adiposity; however, this rarely occurs in isolation. Obesity is often accompanied by a multitude of comorbidities, including hypertension, diabetes mellitus, metabolic syndrome, obstructive sleep apnea, coronary artery disease, and dyslipidemia (Hruby and Hu, 2015). These obesity-related disorders, each with unique pathophysiology, make the investigation of mechanistic links between obesity and its diverse sequelae difficult.

While mechanisms of AF pathogenesis in the context of obesity is an area of ongoing research, progress has been somewhat slowed by the interplay of obesity and related diseases (Karam et al., 2017). Indeed the initiation of the pathogenic pathways leading to AF varies with disease (Goudis et al., 2015). In the context of obstructive sleep apnea, coronary artery disease, and microvascular disease, ischemia is a significant contributor to AF (Tanigawa et al., 2006). However, in the setting of poorly managed diabetes mellitus or metabolic syndrome, increased advanced glycation end products (AGE’s) and receptor (RAGE) upregulation contribute strongly to the pathophysiology of AF in these populations (Kato et al., 2008; Giacco and Brownlee, 2010). In the hypertensive patient, renin-angiotensin–aldosterone system (RAAS) activation and physical stressors on the heart may be the prominent drivers predisposing to AF (Tadic et al., 2014; Goudis et al., 2015).

**FIGURE 1:**
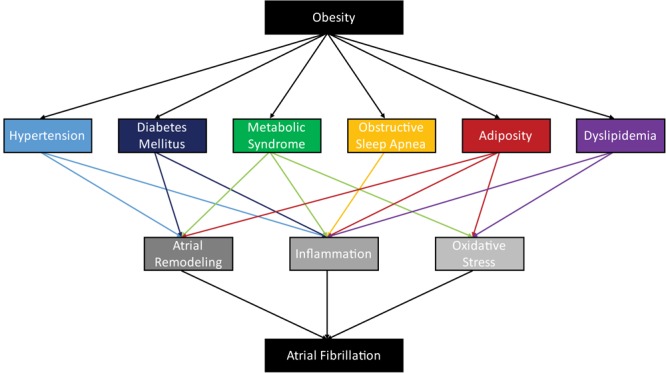
Obesity is associated with a diverse assortment of obesity-related diseases, whose pathophysiologies contribute to the arrhythmogenesis of atrial fibrillation through atrial remodeling, inflammation, and oxidative stress.

However, these diverse pathways converge on key downstream-mediators of AF, which, in turn, contribute to the fulfillment the criteria required for reentrant arrhythmia. AF is driven by the interrelated processes of atrial remodeling, oxidative stress, and inflammation (Figure 1). We assert that focusing on these points of convergence is likely to yield more broadly applicable knowledge and will foster the development of targeted therapies for the treatment of AF.

In obesity and related disorders, atria are remodeled structurally and electrically, providing the substrate for AF (Tse et al., 2016; Karam et al., 2017; Oba et al., 2018). Atrial structural remodeling, including hypertrophy and enlargement, fibrotic infiltration, and fat deposition is a prominent feature of AF and contributes to altered conduction (Jalife and Kaur, 2015; Mahajan et al., 2015). In atrial electrical remodeling, the altered expression and regulation of ion channels, gap junctions, and calcium handling, further contributes to the conduction changes required to sustain AF (Dudley et al., 2005; Lin et al., 2006, Liu et al., 2013). Additionally, obesity induces cardiometabolic changes, that reduce metabolic flexibility and cause increased oxidative stress (Cole et al., 2011; Niemann et al., 2017). Finally, obesity is characterized by inflammation, which leads to increased expression of pro-inflammatory cytokines that prompt further structural and electrical remodeling, contributing to AF pathogenesis (Harada et al., 2015; Hu et al., 2015).

**FIGURE 2:**
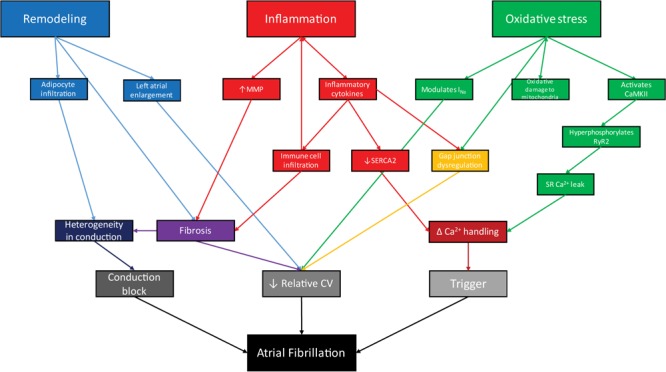
In the context of obesity, remodeling, inflammation, and oxidative stress act through interrelated processes to facilitate arrhythmogenesis via conduction block, reduced conduction velocity, and arrhythmogenic triggers, leading to atrial fibrillation.

While the initiation of pathogenic pathways leading to AF varies with disease, the pathophysiology of AF in obesity is united by shared mediators of altered atrial conduction. In the context of obesity, AF is driven by atrial remodeling, oxidative stress, and inflammation (Figure 2). We suggest focusing on these downstream mediators of AF in obesity is likely to yield more broadly applicable data, including novel therapeutic targets.

## Atrial Remodeling

Obesity and associated diseases are correlated with atrial remodeling (Healey and Connolly, 2003; Lau et al., 2010; Abed et al., 2013a). Atrial remodeling, in turn, directly contributes to the pathophysiology of atrial fibrillation. Atrial remodeling can be split into electrical and structural remodeling.

### Structural Remodeling

In obesity, increases in both fat mass and lean body mass lead to an overall increase in body size. This increase in body size is accompanied by a necessary increase in blood volume. This increase in volume causes stress to the left ventricle, which, if great enough, can cause left ventricular dysfunction increasing afterload, causing increases in atrial volume and pressure. This increase in both volume and pressure contribute to left atrial enlargement (Rosenberg and Manning, 2012). Additionally, hypertension, which is strongly correlated with obesity (Healey and Connolly, 2003; Lau et al., 2010; Abed et al., 2013a), contributes to left atrial enlargement through increased afterload and increased peripheral resistance. Increased atrial size provides a substrate for the development of atrial arrhythmia, including atrial fibrillation (Healey and Connolly, 2003; Pathak et al., 2015).

In addition to causing increased atrial size, obesity also causes tissue remodeling, including increases in fibrosis, fatty infiltration, and pericardial fat deposition. Fibrosis is characterized by the deposition of collagen-rich tissue infiltrating the myocardium. Fibroblasts reside within the interstitium of the myocardium and help maintain homeostasis of the extracellular matrix (ECM). The ECM plays an essential role in maintaining the structure of the of cardiac muscle tissue and, in a network with fibroblasts and myocytes, acts as a sensor of mechanical, electrical, and chemical stimuli (Dzeshka et al., 2015). However, when cardiac damage occurs, as observed in obesity-related stretch or diabetes-related glycosylation, fibroblasts transdifferentiate into myofibroblasts, specialized pro-fibrotic, pro-inflammatory cells. Myofibroblasts occur within the myocardium and can derrive from fibroblasts, vasculature associated smooth muscle, and perivascular pericytes (Squires et al., 2005; Porter and Turner, 2009; Kramann et al., 2015). Myofibroblasts are able to tolerate the increased tensile force within the heart due to increased expression of α-smooth muscle actin and the formation of stress fibers (Shinde and Frangogiannis, 2014). While the myofibroblasts are able to withstand tensile stress, they contribute to the pathologic remodeling observed in AF. First, the deposition of collagen into the ECM by myofibroblasts reduces the compliance of the heart (Travers et al., 2016). Second, increased ECM deposition can interrupt cell-to-cell connections, changing the electrical conductivity of the heart, contributing to the pro-arrhythmic substrate by the relative slowing of conduction velocity. Myofibroblasts can be activated by mechanical strain, cytokines and oxidative stress. TGF-β promotes activation of myofibroblasts by increasing α-SMA expression (Thannickal et al., 2003). Oxidative stress also promotes the transdifferentiation of fibroblasts to myofibroblasts, and treatment with apocynin, and tempol, antioxidants, has been shown to decrease myofibroblast features within cells (Zhao et al., 2008). Additionally, ECM homeostasis is dysregulated through the activation of matrix metalloproteinases (MMPs) and inactivation of tissue inhibitors of metalloproteinases (TIMPs) in obesity, which is mediated in part by oxidative stress (Okamoto et al., 2001; Brown et al., 2004; Donnini et al., 2008; Viappiani et al., 2009). Furthermore, angiotensin, a key player in the RAAS, and TGF-β1, an inflammatory cytokine, are potent stimulators of collagen synthesis (Khan and Sheppard, 2006; Leask, 2010). Together, these disturbances in the ECM cause abnormalities in cardiac contraction, relaxation, and conduction (Tarone et al., 2014; Dzeshka et al., 2015).

Mirroring the general accumulation of adipose tissue, pericardial adipose tissue (PAT) and epicardial adipose tissue (EAT) are increased in obesity. As described below, cardiac-associated adipose tissue has diverse effects, contributing to AF pathogenesis. Pericardial fat volume is correlated with left atrial enlargement and with an increased risk of AF. EAT is highly biologically active, releasing a variety of factors that impact AF progression including inflammatory cytokines (discussed below), and growth and remodeling factors (Lavie et al., 2017). Additionally, adipocytes from EAT can infiltrate directly into the myocardium. This invasion of non-excitable tissue contributes to a decrease in conduction velocity, increasing the potential for arrhythmia.

### Electrical Remodeling

Electrical remodeling, the modulation of cardiomyocyte excitability or electrical activity, is essential to the development of arrhythmia (Abed et al., 2013a; Goette et al., 2016; Gasparova et al., 2017; Oliver et al., 2017). Modulation of the activity of any ion channel that contributes to the cardiac action potential can alter cardiac conduction and contribute to reduced action potential duration, decreased conduction velocity, or dysregulated calcium handling, providing an arrhythmogenic substrate (Gasparova et al., 2017). While data are not homogeneous, rabbit, sheep, and guinea pig models indicate diet-induced obesity may decrease action potential durations, effective refractory periods, L-type calcium current, and conduction velocities (Abed et al., 2013a; Zarzoso et al., 2014; Aromolaran et al., 2016; Srivastava et al., 2018). These reductions are due to electrical remodeling through alterations in gap junctions, ion channel function, and calcium handling.

Gap junctions are composed of connexin dodecamers and form physical and electric connections between cardiomyocytes, allowing for the passage of ions, second messengers and current between cells. Gap junctions allow the conduction of action potentials between cardiomyocytes and facilitate synchronous contraction of cardiac tissue. Obesity causes a shift in the ratio of the two cardiac connexin isoforms, connexin 40 and connexin 43, present in gap junctions (Lin et al., 2006; Hu et al., 2015). Additionally, connexin 43 is hyperphosphorylated and lateralized, slowing longitudinal conduction velocity and potentially contributing to non-uniform conduction.

Tightly regulated calcium signaling is essential for normal excitation and contraction of cardiomyocytes. Through various mechanisms, including alterations in protein expression levels and post-translational modifications, dysregulation of calcium handling contributes to the development and maintenance of AF in obese populations.

Current density through the L-type calcium channel (I_Ca,L_) is generally reduced in atrial fibrillation (Yue et al., 1997; Van Wagoner, 2003; Carnes et al., 2007; Gasparova et al., 2017). Although there is no consensus on the mechanism by which this occurs, oxidative stress may contribute through glutathione reduction and S-nitrosylation (discussed below) (Carnes et al., 2007; Gasparova et al., 2017). In AF, I_Ca,L_ is further reduced through the expression of the brown adipose-related microRNA, miR-328, which reduces the expression of L-type calcium channels (Oliverio et al., 2016). miR-328 targets CACNA1C and CACNB1 mRNA, which encode subunits of the L-type calcium channel. miR-328 is upregulated 3.9-fold in canine models of AF, and about 3.5-fold in AF patients (Oliverio et al., 2016; Gasparova et al., 2017). Furthermore, forced expression of miR-328 caused decreases in I_Ca,L_, which then caused an AF phenotype. While reduction of I_Ca,L_ is protective against intracellular calcium overload, which can cause triggered activity, it does contribute to the arrhythmogenic substrate by causing a relative slowing of conduction velocity (Oliverio et al., 2016; Gasparova et al., 2017).

CaMKII can be activated by a variety of mechanisms, including oxidative stress (discussed below). Once activated CaMKII hyperphosphorylates the cardiac ryanodine receptor (RyR2), causing sarcoplasmic reticulum (SR) leak, which may stimulate delayed afterdepolarizations, triggering calcium-induced calcium release and serving as a trigger for arrhythmia.

A study by Hohl et al. (2017) found that expression of sarcoplasmic reticulum calcium ATPase type 2A (SERCA 2A), was reduced by 36% in obese rats compared to their lean counterparts. SERCA2 functions to return calcium to the lumen of the SR. Decreases in SERCA2 function lead to increased free calcium in the cytosol, permitting asynchronous contraction and contributing to heterogeneity in conduction, which is characteristic of AF (Hohl et al., 2017).

Through a variety of mechanisms including increased atrial size, adipose infiltration, fibrosis, ion channel dysregulation and alterations in calcium handling, obesity and its sequelae contribute to atrial structural and electrical remodeling. Taken together the changes observed in obese hearts predispose to AF by relative wavelength reduction compared to atrial size, causing conduction block, and causing arrhythmogenic triggers.

## Oxidative Stress

In healthy tissue, physiologic levels of reactive oxygen species (ROS) act as essential signaling molecules (Niemann et al., 2017). However, imbalance of oxidants to antioxidants, favoring excess production of or mislocalization of ROS, can contribute to disease pathophysiology. This imbalance, referred to generally as oxidative stress, can be detrimental to cardiac function and contributes to increased risk of AF. ROS refers to any radical or non-radical small molecule that contains oxygen and acts as an oxidant in non-enzymatic reactions (Huang et al., 2009; Gasparova et al., 2017). Examples include, but are not limited to, peroxides, superoxides, nitrous oxide (NO) and singlet oxygen. When present in excess, ROS may cause DNA damage, apoptosis and hypertrophy (Huang et al., 2009). Oxidative stress is increased in obesity-related diseases, including diabetes mellitus, hypertension, and heart failure (Giordano, 2005; Mueller et al., 2005; Pacher and Szabo, 2006).

In obesity, cardiometabolic changes in the heart favor production of ROS. Obese hearts are less metabolically flexible, relying preferentially on fatty acid oxidation (Cole et al., 2011). Due to fuel excess in obesity, circulating levels of free fatty acids and triglycerides are increased. Cardiac fatty acid uptake, in turn, is increased, while glucose uptake and oxidation are reduced (Lopaschuk et al., 2010). Furthermore, accessory glucose metabolism pathways, including the polyol pathway and hexosamine pathway are increased (Kolwicz et al., 2013). These changes result in increased mitochondrial uncoupling due to the increased rate of fatty acid oxidation, accompanied by increased ROS production (Cole et al., 2011). Further, oxygen utilization efficiency is decreased, and relatively less ATP is produced for the amount of fuel oxidized, contributing to further mitochondria dysfunction (Cole et al., 2011; Niemann et al., 2017).

In addition to the altered fuel utilization, oxidative stress occurs in obesity and diabetes mellitus due to non-fatty acid oxidation sources. In hyperglycemia, saccharide self-oxidation and non-enzymatic protein glycosylation through the AGE/RAGE system further increase the production of ROS (Kato et al., 2008; Giacco and Brownlee, 2010). Further, in diabetes mellitus, the internal mitochondrial membrane voltage is increased, further uncoupling the electron transport chain (Boudina et al., 2005). Increased oxidative stress has been shown to interfere with mitochondrial biogenesis and to cause reductions in NADH dehydrogenase levels, especially in young obese subjects propagating oxidative damage (Niemann et al., 2011). Finally, oxidation defense systems that act to reduce ROS and mitigate oxidative damage, are reduced through decreased vitamin availability (vitamin C and E), inactivation of antioxidant enzymes, and down-regulation of glutathione (Anderson et al., 2009; Zhang et al., 2014).

Glutathione, the most abundant endogenous reducing agent, is critical to the maintenance of redox state in the atria. Glutathione depletion is associated with both diabetes and obesity (Keaney et al., 2003; Martin-Gallan et al., 2003). Furthermore, glutathione levels are reduced in AF patients compared to controls, and experimental inhibition of glutathione action decreased atrial I_Ca,L_ density (Carnes et al., 2007). These data indicate the essential role of glutathione in mitigating electrical remodeling that permits AF.

Several studies have indicated oxidative stress in the pathogenesis of post-operative AF (POAF). Right atrial NADPH oxidase activity, serum oxidation markers, and serum peroxidase levels are increased in patients who develop POAF (Ramlawi et al., 2007; Kim et al., 2008; Lubbers et al., 2015; Wu et al., 2015). Additionally, increased total dietary antioxidant capacity has been associated with reduced risk of POAF (Costanzo et al., 2015). While studies in canine models of AF have suggested that ascorbate administration may reduce POAF risk (Carnes et al., 2001), administration of vitamin C for the prevention of POAF in clinical trials has produced mixed results, with studies in the United States failing to show a benefit (Hemila and Suonsyrja, 2017).

Oxidative damage in the obese heart contributes to electrical and structural remodeling observed in obesity, predisposing to conduction changes requisite for atrial fibrillation. Oxidative stress contributes to triggered activity through increased leakiness of the sarcoplasmic reticulum. Reactive oxygen metabolites activate CaMKII, that then contributes to the hyperphosphorylation of RyR2, contributing to electrical remodeling, as described above (Shan et al., 2012; Karam et al., 2017). One study proposed that excess ROS would oxidize amino acid residues within the regulatory domain of CaMKII. This oxidized form, ox-CaMKII, is constitutively active, contributing to electrical remodeling characteristic of AF (Anderson, 2015). Oxidative stress contributes to reduced conduction speeds through both modulation of the sodium current, I_Na_ and, gap junction dysregulation (Liu et al., 2012; Kim et al., 2015). There are diverse reports of connexin 43 dysregulation due to oxidative stress (Karam et al., 2017). Specifically, oxidative stress may contribute to gap junction abnormalities seen in obesity by slowing forward trafficking and causing hyperphosphorylation of connexin 43, as discussed previously, altering conduction through resultant gap junctions, resulting in conduction slowing (Lin et al., 2006; Smyth et al., 2010; Gemel et al., 2017). Oxidative stress contributes to structural remodeling through induction of fibroblast transdifferentiation into myofibroblasts, as well as through activation of MMP’s and down regulation of their inhibitors, TIMPs (Kandasamy et al., 2010). Peroxynitrate directly activates MMP-1,2,8 and 9, while inactivating TIMP-1 and 4, leading to increased fibrosis and dysregulation of ECM homeostasis (Okamoto et al., 2001; Brown et al., 2004; Donnini et al., 2008; Viappiani et al., 2009). Together, this oxidative stress-induced electrical and structural remodeling contributes to the relatively slowed conduction velocity and may contribute to conduction block required for a reentrant arrhythmia, such as atrial fibrillation.

## Inflammation

Obesity is characterized by a constant, low-grade inflammatory state. This inflammation contributes to the pathophysiology of many obesity-related diseases, including AF (Lastra and Sowers, 2013). Acute, induced atrial inflammation in canine models post-operatively caused heterogeneity in conduction and led to increased AF duration (Ishii et al., 2005). Importantly, treatment of the animals with methylprednisolone mitigated these changes, supporting the causative role of inflammation in AF (Ishii et al., 2005). Inflammation contributes to the pathogenesis of AF through direct regulation of cardiomyocyte function and through its contribution to myocardial adipose infiltration and fibrosis, which contribute to conduction abnormalities. Interestingly, atrial inflammation and fibrosis are closely related, sharing many common signaling pathways with each increasing the likelihood of the other. While often caused in part by inflammation, AF causes further inflammation and remodeling causing additional arrhythmia burden, thus contributing to the clinical adage “AF begets AF” (Goudis et al., 2015).

### Systemic Inflammation

Low-grade systemic inflammation in obesity contributes to obesity-related disease pathophysiology, including atrial fibrillation. Many population-based studies have shown circulating levels of biomarkers of inflammation correlate with AF risk and severity. C-reactive protein (CRP) produced primarily in the liver, is released into circulation, and tumor necrosis factor α (TNF-α) produced in many tissues, correlates with AF incidence and severity (Chung et al., 2001; Li et al., 2010). Circulating CRP and TNF-α levels are lowest in patients in sinus rhythm, increased in those with paroxysmal AF, and further increased in patients with persistent AF (Chung et al., 2001; Li et al., 2010). Similarly, circulating IL-10 and local IL-8 in the atria are increased in persistent AF when compared to paroxysmal AF and sinus rhythm (Liuba et al., 2008; Li et al., 2010).

Systemic inflammation also contributes to the incidence of POAF. POAF occurs in up to 16–50% of patients following cardiac surgery (Harada et al., 2015). Preoperative circulating CRP, IL-2, and IL-6 levels have been shown to predict the incidence of POAF (Maesen et al., 2012; Liew et al., 2013). Treatment with colchicine, a suppressor of leukocyte activation through inhibition of microtubule formation, may reduce POAF incidence (Deftereos et al., 2018; Vrachatis et al., 2018). The COPPS study demonstrated colchicine treatment beginning on postoperative day 3 reduced incidence of POAF (Imazio et al., 2011). However, this study did not investigate POAF in the immediate postoperative period. A follow-up study, COPPS-2, which initiated colchicine therapy 48–72 h before surgery, failed to show a reduction in POAF incidence. This may be, in part, due to intolerance of colchicine treatment, as analysis of only subjects with at least 80% adherence to treatment showed reduced POAF incidence (Imazio et al., 2014). Colchicine is also being investigated for reduction of AF recurrence following pulmonary vein isolation by Deftereos et al. (2012, 2014) in two studies treatment with colchicine initiated at the time of or 3 months following ablation reduced AF recurrence rate and increased recurrence-free time. While these studies suggest a potential role for colchicine treatment in AF, other studies have failed to show reduced POAF with colchicine treatment, warranting further investigation (Meurin et al., 2015; Tabbalat et al., 2016; Zarpelon et al., 2016).

### Adipose-Derived Inflammation

In addition to systemic inflammation, inflammation originating from the adipose tissue also contributes to AF pathogenesis. Adipose tissue was once thought of as an inert lipid storage organ. However, it is now understood to be an active endocrine organ, secreting an assortment of cytokines, called adipokines, which can alter the function of other cells in a paracrine or endocrine manner. Adipose tissue function varies with location, with different depots having different secretory phenotypes (Sackmann-Sala et al., 2012; Samanta et al., 2016).

Adipose tissue surrounds the heart, and in healthy states, is cardioprotective, offering mechanical support and cushioning, while acting as a free fatty acid buffer and energy source for the myocardium (Iozzo, 2011; Nagy et al., 2017). Cardiac adipose tissue includes both epicardial adipose tissue (EAT) and pericardial adipose tissue (PAT). While these fat depots are spatially and functionally disparate, these terms are not standardized, and studies may use EAT and PAT interchangeably (Samanta et al., 2016). Due to its direct contact with the myocardium, EAT releases adipokines directly against the myocardium contributing to paracrine inflammation of cardiac tissue and to direct infiltration of adipose tissue into the heart, causing conduction block (Iozzo, 2011). The Framingham Heart Study demonstrated PAT, but not visceral or intrathoracic adipose tissue mass was correlated with increased odds of AF, even after controlling for change in BMI (Thanassoulis et al., 2010). Additionally, studies have shown increased AF chronicity and symptom burden with increasing PAT volume (Wong et al., 2011).

The observed correlations of EAT and PAT with AF are due, in part, to the endocrine function of adipose tissue. EAT releases a variety of biologically active adipokines, which act through autocrine, paracrine, and endocrine pathways. Some adipokines, including adiponectin and omentin, are generally considered beneficial, with anti-inflammatory effects (Lavie et al., 2017). Increased levels of adiponectin secreted from both peripheral and epicardial adipose tissue may offer some protection from arrhythmia, as increased epicardial adiponectin levels pre-operatively have been associated with maintenance of sinus rhythm in surgery (Kourliouros et al., 2011). Alternatively, metabolically deregulated obese adipose tissues released a variety of inflammatory cytokines contributing to the pathophysiology of obesity-related dysfunction. EAT has been shown to secrete CRP, TNF-α, IL-2, IL-6, IL-8, and monocyte chemoattractant protein-1 (MCP1), among others (Lavie et al., 2017). These cytokines act through diverse mechanisms to alter cardiac function and predispose to disease through cellular infiltration, fibrosis, and electrical remodeling. MCP1 causes the infiltration of macrophages and neutrophils into the myocardium, as has been shown in atrial samples from patients with AF (Frustaci et al., 1997; Chen et al., 2008; Yamashita et al., 2010). The infiltrating neutrophils secrete myeloperoxidase (MPO), an oxidizing agent (Rudolph et al., 2010; Harada et al., 2015). Through oxidative stress, MPO causes fibrosis of the myocardium, contributing to conduction abnormalities and predisposing to AF.

Fibrosis is a common result of inflammatory signaling in the heart, mediated by inflammatory cytokines. TNF-α is upregulated in obesity and contributes to fibrosis through the upregulation of matrix metalloproteinases (MMPs) and the modulation of ECM degradation (Liew et al., 2013). Transforming growth factor beta (TGF-β) family proteins are secreted at increased levels in obese EAT leading to increased fibrosis and inflammation (Nattel and Harada, 2014). Activin A, a member of the TGF-β family, directly causes the deposition of fibrotic material, as demonstrated in cell culture. This reaction was mitigated by the addition of an anti-activin A antibody, indicating causation and supporting the direct role of inflammatory cytokines in cardiac fibrosis (Yndestad et al., 2004). This fibrosis, in turn, slows cardiac conduction, predisposing to arrhythmia.

Inflammatory cytokines, especially TNF-α, also contribute to the electrical remodeling of the atria likely contributing to the observed correlation of inflammatory cytokine levels with AF (Hu et al., 2015). Experiments in rabbits have shown that increases in TNF-αcause altered calcium handing in cardiomyocytes near the pulmonary vein, leading to arrhythmia (Lee et al., 2007). This may be due to methylation of the promoter of sarcoplasmic reticulum Ca2+-ATPase 2 (SERCA2), causing decreased expression (Kao et al., 2010). Further, in mouse models of TNF-α overexpression, connexin 40 and 43 were downregulated and lateralized, respectively, causing impaired conduction (Sawaya et al., 2007). Finally, cardiac-specific overexpression of TNF-α increased atrial arrhythmias, suggesting a causative role for inflammatory cytokines in the electrical remodeling leading to AF (Sawaya et al., 2007; Hu et al., 2015).

### Hypoxia-Induced Inflammation

In obesity, there are many sources of transient and persistent hypoxia, including obstructive sleep apnea, coronary artery disease, and microvascular disease. Because hypoxia can be a strong driver of inflammation (Imtiyaz and Simon, 2010; Eltzschig et al., 2014), it is likely this hypoxia contributes to the inflammatory phenotype observed in obesity through hypoxia-inducible factor 1α (HIF-1α), which acts in hypoxia to increase vessel permeability leading to increased immune cell extravasation (Imtiyaz and Simon, 2010). The constant low-grade systemic inflammation characteristic of obesity, along with the pro-inflammatory secretome from adipose tissue and hypoxia-induced inflammatory signaling cause increased inflammation in the atria, directly contributing to atrial structural and electrical remodeling, facilitating AF.

## Therapies

Current therapies are used to manage AF include rate control, rhythm control, and ablation. However, these treatments are accompanied by side effects that limit their use, highlighting the need for more targeted therapies for AF.

### Pharmacologic Therapy

β-blockers are used in 60–70% of patients with persistent or permanent AF for rate control (Van Gelder et al., 2010; Camm et al., 2011; Dorian and Angaran, 2014). However, aggressive rate control with β-blockers (<80 beats per min) is associated with increased risk of adverse effects and worsening quality of life, when compared to more conservative management (Groenveld et al., 2013). Non-dihydropyridine calcium-channel blockers including verapamil and diltiazem, which control ventricular rate by slowing conduction through the atrioventricular node, have been shown to be more effective at controlling AF symptoms than β-blockers (Rodriguez Padial et al., 2016). Digoxin, a cardiac glycoside, acts mainly by inhibiting the Na^+^/K^+^ exchanger leading to decreased heart rate and increased force of contraction. However, Digoxin is not generally a first-line treatment due to the risk of ventricular arrhythmia (Rodriguez Padial et al., 2016).

Rhythm control can also be used in the management of AF, but it carries a higher risk for complications. Cardioversion can be accomplished by two methods: electrical or pharmacological. Electrical cardioversion requires a trans-thoracic shock. While the exact mechanism by which this terminates the AF is not known, studies have postulated that depolarization of a critical mass or prolongation of the atrial refractory period could prevent reentrant currents (Zipes et al., 1975; Ideker et al., 2000). Pharmacologic cardioversion utilizes sodium or potassium channel blockers to depolarize cardiomyocytes and increase refractoriness. However, class IA Sodium channel blockers are contraindicated in patients suffering from structural heart disease, commonly encountered in obese patients, as it was shown to increase mortality (Echt et al., 1991).

The lack of sufficient management of AF with pharmacology ion channel modulating drugs elucidates a significant need for novel targeted therapies against non-ion channel targets.

### Ablation

While pharmacological agents remain common treatments for AF, catheter ablation of the arrhythmogenic path is also an effective therapy. Several studies have shown ablation is associated with improved outcomes 12-months following ablation (Wazni et al., 2005; Oral et al., 2006; Stabile et al., 2006). While this treatment can be useful, it is dependent on the skill of the clinician and has a ∼3% risk of major complications. Furthermore, recent studies have suggested potential late-recurrence of AF among other compilations (Ouyang et al., 2010).

While ablation remains an effective therapy, post procedural recurrence of AF is a common problem. Studies have shown that ablation is more effective at lower BMIs. Jongnarangsin et al. (2008) demonstrated that patients were 5% more likely to experience recurrence for each single point increase of BMI. This increased likelihood is due, in part, to obesity-related diseases, including OSA and hypertension, which increase the risk of recurrence post-ablation (Jongnarangsin et al., 2008; Hohl et al., 2017). Further, increased pericardial fat is predictive of recurrence of AF after ablation. Finally, there have been studies suggesting reduced AF recurrence following treatment with the anti-inflammatory medication, colchicine, highlighting inflammation as a potential contributor to post-ablation AF recurrence (Deftereos et al., 2012, 2014). These data suggest that without addressing obesity, related pathologies, and their downstream mediators, atrial remodeling, inflammation, and oxidative stress, will continue to provide an AF substrate, leading to AF recurrence.

### Weight Loss

Some studies have found that aggressive risk factor management, including weight loss, can dramatically improve incidence of recurrence, even in the presence of secondary pathologies (Pathak et al., 2014; Hohl et al., 2017). A randomized control study demonstrated that weight loss intervention resulted in a significant decrease in both atrial fibrillation symptom burden and symptom severity (Abed et al., 2013b). As obesity is closely associated with other pathologies that contribute to AF risk, including hypertension, obstructive sleep apnea, and diabetes, weight loss is associated with improvement of related diseases.

Multiple studies of the effect of weight loss on cardiac structure have shown a decrease in left ventricular mass (Alexander and Peterson, 1972; Backman et al., 1979; Karason et al., 1997). A decrease in weight reduces total and central blood volume, therefore, relieving ventricular stress by reducing afterload. A decrease in weight proportionally lowers cardiac output through decreasing total blood volume (Alexander and Peterson, 1972; Backman et al., 1979; Karason et al., 1997). Indeed, bariatric surgery has been reported as the best intervention to reduce AF risk and symptom burden in obesity, due to the extensive amount of weight loss (Alpert et al., 2014). As previously discussed, cardiac fibrosis and hypertrophy are common in obesity. While the hemodynamic stress can be alleviated with weight loss alone, the fibrotic and hypertrophic effects are more permanent. Additional therapies are needed to manage additional cardiac consequences of obesity.

### Targeted-Therapies

Considering the prevalence of AF and the complications and side effects associated with current AF treatments, there is a need for new, targeted therapies. Due to the apparent role of inflammation and oxidative stress in the pathophysiology of AF in obesity, investigation of drugs that modulate these pathways may yield promising new therapeutic strategies. Due to the risk for arrhythmia with ion-channel modulation, non-ion channel proteins are attractive potential drug targets. As discussed above, CaMKII hyperphosphorylates RyR2 causing Ca^2+^ leak and promotes late Na^+^ release, lengthening action potential duration, promoting arrhythmia (Hund et al., 2010; Grandi and Dobrev, 2017). AS105, an ATP-competitive inhibitor of CaMKII has been shown to improve cardiomyocyte function (Neef et al., 2018).

Thiazolidinediones (TZDs), peroxisome proliferator-activated receptor gamma activators, are a class of drugs initially implemented in the treatment of diabetes, as they act as insulin sensitizers and reduce inflammation. While TZDs are used only as a second-line treatment for diabetes, they may offer anti-arrhythmic benefits. A sizeable retroactive study of Danish population affected by diabetes showed that patients who were prescribed TZD were 24% less likely to develop arrhythmia as compared to those who took other second-line drugs and 31% less likely than those who did not take any secondary treatment (Pallisgaard et al., 2017). Further exploration of TZDs as anti-arrhythmic agents would be valuable.

Statins are used to treat cardiovascular disease through the prevention of the formation of cholesterol. Statins competitively bind to HMG-CoA reductase and inhibit the enzyme, so that it is unable to convert Acetyl-CoA to mevalonate, a progenitor of cholesterol. Statins also reduce inflammation and can reduce the production of ROS via the inhibition of NADPH oxidase activity within cardiomyocytes. While a trial of the use of statins to treat existing arrhythmia did not yield significant results, they remain promising for the prevention of arrhythmia (Reilly et al., 2011).

## Conclusion

Atrial fibrillation is the most common cardiac arrhythmia and is associated with reduced quality of life, heart failure, stroke, and increased mortality (Camm et al., 2012; Hu et al., 2015). Almost 20% of AF cases may be attributable to overweight or obesity (Huxley et al., 2011). To sustain AF, a reentrant arrhythmia, three basic criteria must be met: (1) the atria must be sufficiently electrically or structurally remodeled such that the tissue ahead of the wavefront is excitable through reduced conduction velocity increased total path length, and/or decreased effective refractory period. (2) There must be unidirectional conduction block, which prevents wave fronts from meeting each other and self-extinguishing. (3) There must a unexcitable obstacle around which the wave rotates. Heterogeneous obesity-related diseases, including obstructive sleep apnea, diabetes mellitus, metabolic syndrome, coronary artery disease, and dyslipidemia, contribute to the establishment of these criteria. While the upstream pathophysiologic pathways that facilitate the contribution of each of these obesity-related diseases to AF differ, there is a convergence upon three mediators of AF: atrial remodeling, oxidative stress, and inflammation. These three mechanisms do not act independently, but are interrelated, creating a self-perpetuating cycle. This likely contributes to the progressive nature of AF clinical progression and the clinical adage “atrial fibrillation begets atrial fibrillation” (Tsang et al., 2008; Karam et al., 2017).

Recent studies have highlighted the role of genetics in AF development (Chen et al., 2003; Wilde and Bezzina, 2005; Olson et al., 2006; Ellinor et al., 2008; Roberts and Gollob, 2014). Variable penetrance of AF-associated variants reflects the complexity of the pathogenesis of AF. Furthermore, with the complex interplay of obesity and the development of AF, it is likely that, given the effects of obesity on cardiac structural and electrical remodeling, that obesity could affect the penetrance of AF susceptibility variants. This relationship is not well understood and is an avenue for future research.

While the pathogenesis and pathophysiology of AF are complex, the contribution of obesity and obesity-related comorbidities to AF highlights the potential benefit of weight-loss as an AF management strategy. As obesity causes structural and electrical remodeling, oxidative stress and increases systemic and local inflammation, weight-management represents a broad treatment that directly addresses several central mediators AF.

Mirrored by increasing global obesity rates, rates of AF are rapidly increasing. Some predictions indicate the prevalence of AF will nearly triple in the next 30 years, reaching epidemic proportions (Morin et al., 2016; Lavie et al., 2017). Limitations of current AF therapies combined with increasing prevalence highlight the need for new therapeutics to treat AF. We suggest that therapeutic strategies targeting remodeling, oxidative stress and inflammation will result in more successful, safer, and more broadly applicable treatments.

## Author Contributions

EL and PM conceived the manuscript. EL and MP wrote the first draft of the manuscript. PM critically reviewed and revised the manuscript.

## Conflict of Interest Statement

The authors declare that the research was conducted in the absence of any commercial or financial relationships that could be construed as a potential conflict of interest.
